# Intraoperative Pain Management for Treatment-Resistant Complex Regional Pain Syndrome: A Case Report

**DOI:** 10.7759/cureus.72935

**Published:** 2024-11-03

**Authors:** Catherine R La Spina, Patricia Pozo, Gisele J Wakim

**Affiliations:** 1 Anesthesiology, Jackson Memorial Hospital, Miami, USA; 2 Anesthesiology, University of Miami, Miami, USA

**Keywords:** chronic neuropathic pain, complex regional pain syndrome, crps pathophysiology, intraoperative ketamine, stages of crps

## Abstract

Complex regional pain syndrome (CRPS) is a chronic neuropathic pain disorder often following trauma, associated with severe pain and autonomic disturbances in the affected limbs. Managing CRPS is challenging due to the lack of FDA-approved medications, often requiring off-label treatments. Traditional options like nonsteroidal anti-inflammatory drugs (NSAIDs) and corticosteroids show limited efficacy, while adjunctive treatments such as gabapentin, antidepressants, and bisphosphonates are increasingly favored. Surgical interventions, including nerve blocks and spinal cord stimulation, may help in refractory cases but have varying success rates. Recent discussions highlight intraoperative ketamine, which targets N-methyl-D-aspartate (NMDA) pathways linked to CRPS. This case study illustrates the complexity of CRPS management, particularly how psychosocial factors and secondary trauma can exacerbate or alleviate symptoms. The case centers on a treatment-resistant flare-up of CRPS, managed through revision neurolysis of the sciatic, tibial, and perineal nerves, along with the release of the right tibial nerve and intraoperative ketamine. Trauma’s impact is evident, as the patient initially went into remission after nerve decompression, only for symptoms to return severely following a subsequent trauma. This emphasizes the need for a multipronged treatment approach. Intraoperative ketamine provides rapid pain relief during and after surgery, benefiting patients with severe chronic pain while reducing post-surgery opioid needs and minimizing dependency risks. Patients typically achieve improved functional recovery and better rehabilitation engagement. Research suggests ketamine may offer long-lasting pain relief and psychological benefits, positively impacting mood and anxiety.

## Introduction

The understanding of complex regional pain syndrome (CRPS) has evolved over time and thus is referred to in the literature under varying terminology that now falls under the umbrella of CRPS. Alternative names include reflex sympathetic dystrophy, algodystrophy, causalgia, Sudeck atrophy, transient osteoporosis, and acute atrophy of bone [1]. The current definition of CRPS describes an array of painful conditions that are characterized by a continuing (spontaneous and/or evoked) regional pain that is disproportionate in time or degree to the usual course [2]. The pain is regional (not in a specific nerve territory or dermatome) and usually has a distal predominance of abnormal sensory, motor, sudomotor, vasomotor, and/or trophic findings. Two types of CRPS are recognized [1]. Type I occurs when there is no confirmed nerve injury and type II occurs when there is known associated nerve injury, confirmed by nerve conduction testing. Type 1 CRPS accounts for approximately 90% of CRPS cases [3].

The Budapest criteria for the clinical diagnosis of CRPS, established by the International Association for the Study of Pain, exhibit a sensitivity of 99% and a specificity of 68% for CRPS [2]. The Budapest criteria require persistent pain that is disproportionate to the inciting event, with sensory, vasomotor, sudomotor, and trophic abnormalities. The expected symptoms include allodynia, skin color and temperature changes, edema, sweating, sensory abnormalities, and motor dysfunction.

In cases where clinical features are atypical, adjunctive diagnostic testing is used to support the diagnosis of CRPS and exclude alternative causes of symptoms. Triple-phase bone scintigraphy (TPBS) is useful for detecting alterations in bone metabolism in patients with CRPS exhibiting active bone resorption [4]. Plain film radiography, while low sensitivity, can also support the diagnosis of CRPS if there is patchy bone osteoporosis involving the affected limb [5]. Finally, different forms of autonomic testing have been utilized for the evaluation of patients with suspected CRPS, specifically the resting sweat output (RSO), the resting skin temperature (RST), and the quantitative sudomotor axon reflex test (QSART) [5]. Of note, a meta-analysis comparing diagnostic imaging in the evaluation for Type I CRPS found that TPBS had a significant advantage over both MRI and plain film radiography in terms of sensitivity and negative predictive value [6]. However, the specificity and positive predictive values showed no statistical significance between the three imaging techniques.

CRPS profoundly affects patients’ quality of life and daily activities, making effective and timely pain management crucial. The disease may remain chronic for an extensive period of time or resolve completely. It can also recur after a disease-free period, often followed by further trauma or another operation [7]. While there is no “gold standard” for the treatment of CRPS, current management includes the use of oral corticosteroids, anticonvulsants, opioids, bisphosphonates, and analgesic antidepressants [8,9]. More intrusive interventions such as nerve blocks, spinal cord stimulation (SCS), or sympathectomy have also been deemed effective for some patients [8].

## Case presentation

In this case, a 49-year-old obese male with a past medical history of anxiety, depression, sciatica, and hyperlipidemia presented with persistent right calf swelling and pain that eventually led to weakness in dorsiflexion and plantarflexion of his right foot. In September 2020, he reported suffering from an accident while jumping into an above-ground pool. Over the following days, the pain and swelling subsided. However, several weeks later, while cycling, he began experiencing excruciating pain in his right posterior calf, which progressed to encompass his entire foot. Despite attempting to overlook the discomfort, it escalated to a debilitating level by December 2020, prompting him to seek medical evaluation. Interestingly, he also began experiencing pain in his left leg in a similar distribution, albeit less severe than on the right side. Consequently, his pain became so incapacitating that he ceased working in April 2021. Despite conservative management, including medications like pregabalin and baclofen, his symptoms persisted, leading to a neurolysis procedure in March 2022, which provided no relief.

**Figure 1:**
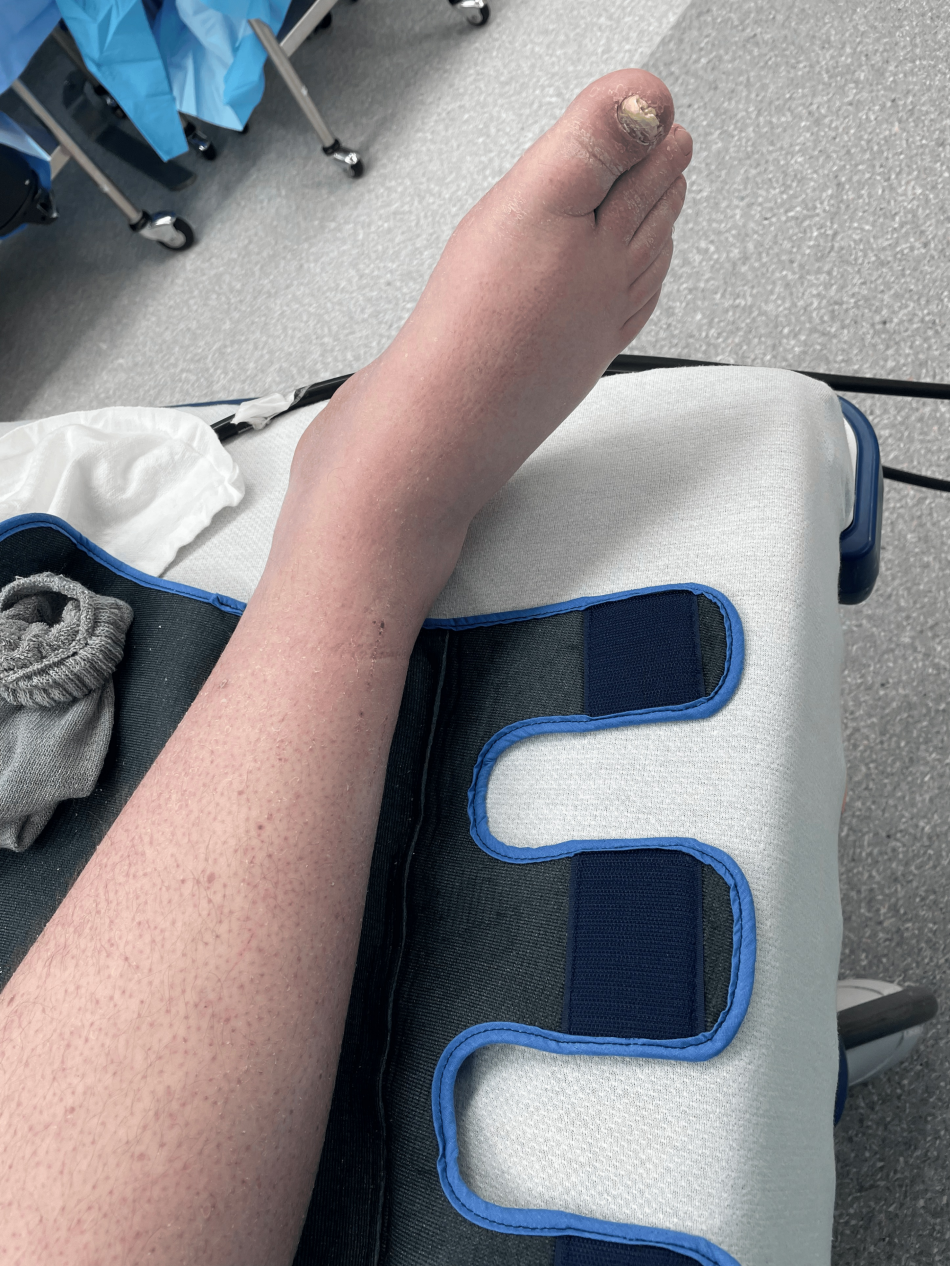
Skin and nail changes of the right lower extremity in February 2024

By October 2022, he sought further care at the Spine and Brain Care at the University of Miami, where imaging revealed fascial scarring and a narrowed tibial nerve, but normal MR signal intensities. Following extensive evaluation, he underwent decompression surgery for the right tibial nerve in March 2023, which initially resulted in significant pain relief. Unfortunately, just two days post-surgery, a traumatic right knee injury from a basketball led to the return of severe, preoperative symptoms. Despite being on medications like Lyrica and oxycodone, he reported a resurgence of debilitating symptoms described as severe pain in the bilateral lower extremities, with the right side being notably worse accompanied by numbness, bilateral foot burning, and redness and swelling in both legs (Figure 1).

**Figure 2:**
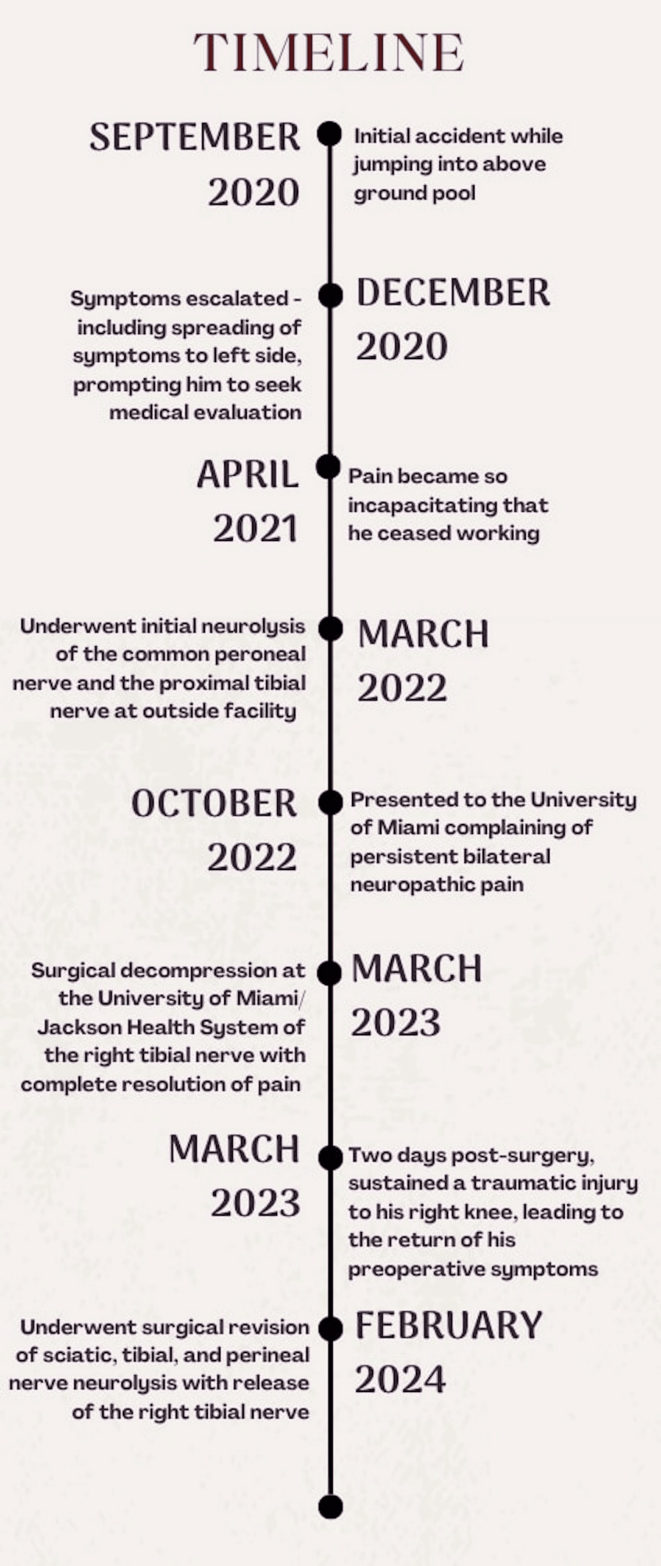
Timeline of events

In response to the recurrence of these troubling symptoms, a revision surgery was scheduled for February 2024, focusing on sciatic, tibial, and perineal nerve neurolysis accompanied by simultaneous release of the right tibial nerve. During this procedure, IV ketamine was introduced for intraoperative pain management due to its efficacy in treating chronic pain syndromes and its potential to provide relief when traditional analgesics fail. This decision was based on the patient’s history of severe pain and the need for a comprehensive approach to pain management during the revision procedure, aimed at enhancing recovery and ameliorating the persistent debilitating symptoms. Refer to the timeline (Figure 2) for the progression of events.

## Discussion

Currently, there is no medication that is marketed specifically for CRPS by the US Food and Drug Administration (FDA), nor is there a single drug or a combination that is guaranteed to be effective for all patients [8]. Thus, the management of CRPS is complex and difficult, primarily consisting of treatment regimens with off-label medications. Initially, conservative measures are preferred with physical therapy, occupational therapy, psychosocial and behavioral management if applicable, and symptomatic pain management with oral medications [8]. Nonsteroidal anti-inflammatory drugs (NSAIDs) and corticosteroids were regarded as traditional medication options; however, recent studies have shown no benefit in support of NSAIDs and the chronicity of CRPS makes long-term corticosteroid usage unfavorable [9,10]. Bisphosphonates have shown positive outcomes for the management of CRPS, with multiple randomized controlled trials (RCTs) demonstrating both safety and efficacy for patients [10,11]. Adjunctive medications for neuropathic pain, such as gabapentin, pregabalin, or a tricyclic antidepressant are also frequently prescribed, specifically gabapentin due to its favorable safety profile [10].

The patient in this case tried and failed conservative management, and continued to have debilitating pain. Several options are available for patients who require additional symptomatic pain management. Interventional and surgical procedures that have been shown to relieve pain in CRPS include nerve blocks, SCS, peripheral nerve stimulation (PNS), and sympathectomy [8,10]. For our specific case, SCS did not show to be beneficial, as the patient reported no improvement of symptoms, and the implant was removed. Undergoing surgery for decompression of the right tibial nerve ultimately provided the patient with the most significant clinical improvement and complete resolution of preoperative pain. Unfortunately, his subsequent injury brought back the pain with worsening severity, necessitating him to undergo revision of the sciatic, tibial, and perineal nerve neurolysis with the release of the right tibial nerve.

During the patient’s last neurolysis procedure, intraoperative IV ketamine was used for pain management, which has been a recent topic of discussion for the management of CRPS. In addition to typical intraoperative pain management with acetaminophen 1000mg and fentanyl 200mcg, the patient was given ketamine 50 mg IV. Ketamine works by targeting N-methyl-D-aspartate (NMDA) nociceptive pathways, which is relevant in the treatment of CRPS as it downregulates the inflammatory response as well as providing analgesic and antidepressant properties [12]. Given its rapid antidepressant effects and enhanced cognitive flexibility over time, this is relevant for cases of CRPS due to the established association with physiatric disease [7]. Multiple studies have shown that the use of ketamine infusions has provided pain relief and induced remission in treatment-resistant CRPS cases, however, systematic reviews have indicated overall low-quality evidence [10,12]. Ketamine has been shown to suppress proinflammatory cytokines in various clinical settings, including major surgery and treatment-resistant depression, potentially explaining its analgesic effects in conditions like CRPS due to its influence on the neuroimmune system [13]. Current literature suggests that approximately one-half of patients with CRPS will experience long-term pain relief from a single ketamine infusion [13]. Notably, the use of ketamine in cases of CRPS could have a positive influence on reduced opioid usage.

While the exact cause of CRPS is not fully understood, it is considered a multifactorial condition with numerous predispositions and risk factors - of which the patient did have relevant risk factors. A recent study of 1043 patients with CRPS found the most common primary causes were fractures (42%), blunt traumatic injuries excluding fractures (e.g., sprains) (21%), surgery (12%), and carpal tunnel syndrome (7%), while 7% had no clear precipitating event [10,14]. One of the hypothesized predispositions includes “psychological instability” (emotionally unstable, nervous, depressive, insecure, anxious, chronic complainers), however, this specific predisposition has been rejected by numerous authors and remains a debated topic [7]. It is also suggested that age and gender play a role in the development of the syndrome, with CRPS having a higher incidence in women and the elderly [7]. There has been a loose association with genetic risk factors, specifically among the human leukocyte antigen (HLA) family. It has been found that the expression of HLA-DQB1 was increased among CRPS patients [9]. It has also been suggested that there is an increased incidence of CRPS in cases of inadequate anesthesia during fracture reduction and poor pain relief during rehabilitation [7,15].

Our patient had a significant history of anxiety and depression requiring pharmacological intervention and regular clinical follow-up. As mentioned above, one of the conservative management options for CRPS is the treatment of any underlying psychosocial and behavioral conditions. Additionally, he had two instances of trauma to the affected limb, a known primary risk factor for the development of CRPS. The original insult was when he jumped into an above-ground pool, with the pain in the right lower limb being exacerbated by cycling in the days following the accident. Furthermore, delayed diagnosis and treatment initiation of CRPS have been regarded as a negative prognostic factor with longer pain duration [16].

Early reports once delineated three sequential clinical stages of CRPS. However, this concept has largely fallen out of favor among experts due to insufficient evidence supporting distinct stages [17]. Stage I, the acute phase, typically manifests with severe limb pain, often following an event or emerging spontaneously and lasting up to three months, and features burning, throbbing sensations, diffuse aching, sensitivity to touch or cold, and localized edema [18]. In stage I, treatment is typically more conservative with NSAIDS, physical therapy, bisphosphonates, and steroids. stage II, the dystrophic phase, sees a progression of the symptoms, with increased soft tissue edema, accompanied by trophic changes of the skin, nails, and hair, soft tissue thickening, and muscle wasting, lasting three to six months. The most severe and chronic stage, stage III atrophic phase, is characterized by reduced mobility, muscle contractures, waxy trophic skin changes with ulceration, and diminished reflexes. Most significantly, severe demineralization is evident in bone radiographs when in stage III [18]. Given that our patient had significant skin and nail changes, without frank ulceration, and the chronicity of his symptoms, he was likely in stage II of CRPS at the time of the procedure. There could be arguments made for stage III given the refractory symptoms spanning over years that had to undergo invasive procedures, but the absence of significant bone demineralization and lack of muscle atrophy argues against stage III.

This patient did experience resolution of symptoms and subsequent remission following decompression of the right tibial nerve. However, he had a second traumatic insult to the right leg, when his right knee was struck by a basketball two days post-operation. One of the notable features of this case is his complete remission followed by a flare-up after injury with the same neuropathic presentation but with worse severity than before. A proposed mechanism for CRPS is an abnormal inflammatory response, specifically an inflammatory response exaggerated in magnitude and without resolution following tissue healing [19]. This abnormal response can spread to contiguous tissue, and secondary trauma can act to facilitate this spread [19], which helps explain the presentation of this patient. The presence of new trauma preceding the onset of CRPS has been previously reported, with one study finding contralateral spread in 37% of patients, ipsilateral spread in 44% of the patients, and diagonal spread in 91% of patients [20]. In the case of this patient, he began experiencing pain in his contralateral (left) leg prior to the occurrence of his secondary trauma. This disease presentation in the contralateral leg is well established, independent of secondary injury, with the spontaneous spread of CRPS from limb to limb being twice as likely to happen contralaterally than ipsilaterally [20]. The hypothesized mechanism for this is that the inflammatory response contains a neurogenic component that is partly under CNS control [19], however, more research is needed on the matter.

## Conclusions

This case focuses on the presentation and management of a treatment-resistant flare-up of CRPS that was ultimately managed with a revision of sciatic, tibial, and perineal nerve neurolysis with the release of the right tibial nerve and usage of intraoperative ketamine. CRPS has many risk factors including psychosocial factors and physical health metrics, many of which are demonstrated in this case. Trauma specifically is a significant risk factor for CRPS - highlighted in our patient as they went into remission following decompression of the right tibial nerve, but symptoms returned with more severity after a secondary trauma to the same limb. Complex cases of CRPS that demonstrate a remitting and relapsing pattern therefore necessitate a multipronged treatment approach, and intraoperative ketamine has emerged as a promising option. The rapid pain relief it provides during and after surgery is particularly beneficial for patients with severe chronic pain and can decrease the reliance on opioids, both intra and post-operatively. In turn, decreased opioid use helps reduce medication side effects and dependency. Ketamine use can also help promote improved functional recovery and pain management allowing for better participation in rehabilitation. Additionally, research suggests that ketamine may offer long-lasting pain relief and psychological benefits including improvements in mood and decreased anxiety. Overall, the use of intraoperative ketamine for managing CRPS is an under-explored area in current literature. Further studies should be aimed at better understanding the pathophysiology of CRPS, as well as continued research on alternative medication options such as ketamine and methadone for treatment-resistant cases.
